# High Temperature CO_2_ Capture Performance and Kinetic Analysis of Novel Potassium Stannate

**DOI:** 10.3390/ijms24032321

**Published:** 2023-01-24

**Authors:** Ross Baird, Ribooga Chang, Ocean Cheung, Aimaro Sanna

**Affiliations:** 1 Institute of Mechanical, Process and Energy Engineering, School of Engineering and Physical Sciences, Heriot-Watt University, Edinburgh EH14 4AS, UK; 2 Nanotechnology and Functional Materials Division, Department of Materials Science and Engineering, The Ångström Laboratory, Uppsala University, 752 37 Uppsala, Sweden

**Keywords:** CO_2_ capture, high temperature sorbents, adsorption, potassium stannate, temperature swing

## Abstract

For the first time, the use of stannate-based sorbents was investigated as high temperature CO_2_ sorption to evaluate their potential to contribute towards reducing carbon emissions. The sorption capacity and kinetics of commercial tin oxide, sodium, potassium and calcium stannates and lab synthesised potassium stannates were tested using thermogravimetric analysis. Commercial K_2_SnO_3_ was found to possess the largest CO_2_ uptake capacity (2.77 mmol CO_2_/g or 12.2 wt%) at 700 °C, which is among the highest for potassium sorbents, but the CO_2_ desorption was not successful. On the contrary, the in-house synthesised K-stannate (K-B) using facile solid-state synthesis outperformed the other sorbents, resulting in a CO_2_ uptake of 7.3 wt% after 5 min, an adsorption rate (0.016 mg/s) one order of magnitude higher than the other stannates, and stability after 40 cycles. The XRD and XPS analyses showed that K-B contains a mixture of K_2_SnO_3_ (76%) and K_4_SnO_4_ (21%), while the Scherrer crystal sizes confirmed good resistance to sintering for the potassium stannates. Among the apparent kinetic model tested, the pseudo-second order model was the most suitable to predict the CO_2_ sorption process of K-B, indicating that chemical adsorption is dominant, while film-diffusion resistance and intra-particle diffusion resistance governed the sorption process in K-B. In summary, this work shows that solid-state synthesised potassium stannate could be an effective sorbent for high temperature separation, and additional work is required to further elucidate its potential.

## 1. Introduction

The largest driver towards climate change has been the increase in greenhouse gasses over the past century, with the most abundant being carbon dioxide. Global atmospheric CO_2_ levels today are the highest they have been in the past 800,000 years with them currently 50% higher than pre-industrial levels at 421 parts per million recorded in 2022 at the NOAA’s Mauna Loa Atmospheric Baseline Observatory [1]. In the attempt to revert this trend, Carbon Capture and Storage (CCS), which allows carbon to be separated and sequestered to prevent it from being released into the atmosphere, has been proposed, among other technologies. However, it has to be said that CCS deployment must increase dramatically from present levels, since if the current deployment rates are projected to 2050, they will cover just 10% of what is required [2]. There are several methods that can be used to separate CO_2_ in order for it to be captured and stored, including absorption, adsorption, and membrane separation. Solid sorbents are viewed as one of the more favourable forthcoming CO_2-_separating materials for pre- and post-combustion processes in a wide range of temperatures (25–700 °C), due to their promising stability and low energy costs to implement and operate compared to the more widely used liquid amines [3,4,5]. Selecting a sorbent for high temperature CO_2_ capture depends on several criteria (selectivity of CO_2_, adsorption and regeneration capacity, kinetics, mechanical strength, tolerance to impurities, and cost) to ensure the operational requirements of the process are met whilst being economically viable [6]. The majority of the research into high temperature CO_2_ sorbents involves using CaO, silicates and zirconates such as sodium zirconate and lithium-based sorbents, due to their known ability to separate CO_2_ from flue gas at high temperatures around the range of 500–700 °C [7,8]. Calcium-based sorbents have been shown to operate successfully at temperatures above 600 °C with calcium oxide (CaO) being one of the most researched due to its low-price natural availability. Unfortunately, CaO has three severe limitations, including slow kinetics after the initial carbonation step (due to product layer formation), energy intensive regeneration, and the requirement for a large surface area [9]. Li- and Na-based zirconates have shown capture capacities of between 0.187 and 0.211 g CO_2_ g sorbent^−1^, which is roughly 80–90% of their theoretical maximum [10]. These results were obtained under a low partial pressure of CO_2_ equal to 0.1 bar, highlighting the material can be used in post-combustion settings. The CO_2_ capture mechanism for lithium zirconate (Equation 1) shows that during the adsorption process, CO_2_ molecules react with lithium zirconate to form a lithium carbonate shell on the surface of the zirconium oxide shell, which covers the unreacted lithium zirconate core. The carbonation continues after the Li_2_CO_3_ and ZrO_2_ shells are produced by CO_2_ and Li^+^ and O^2−^ ions diffusion through the product layer [11].

(1) $$Li_{2}ZrO_{3}+CO_{2}\;⇌\;Li_{2}CO_{3}+ZrO_{2}$$

Although current research and development into CO_2_ High Temperature (HT) sorbents is mainly focused on Ca-, Li-, and Na-supported materials [6,9,12], potassium supported on Al_6_Si_2_O_13_, CaSiO_3_, ZrSiO_4_, ZrO_2,_ and porous carbon have also been considered, with the latter resulting in the highest CO_2_ uptake (9–12 wt%) amongst supported K_2_CO_3_ sorbents [13,14].

Tin oxide (SnO_2_) has a rutile-type structure and is commonly used in various applications such as ceramics, plastics, batteries, and catalysts. It has been shown to have good thermal stability with it being stable in air at temperatures beyond 1400 °C [15]. Stannates and manganates have potential for adsorption of CO_2_ at high temperatures [16]. This is due to the fact that they share a similar ion distribution and molecular structure as zirconates, suggesting that they may share some of the key characteristics that are important for high temperature CO_2_ capture [17]. Both materials have central alkali-based metal with a double-bonded oxygen and two single-bonded oxygen atoms. This closeness in structure indicates stannates will react with CO_2_ under similar adsorption/desorption mechanisms as shown above for lithium zirconate. In addition, the study of the crystalline shape of two oxides, K_2_ZrO_3_ and K_2_SnO_3,_ showed that both have similar base edge-shared MO_X_ square pyramidal structure [17]. Manganates such as Li_2_MnO_3_ have been shown to have a similar structure to that of Li_2_SnO_3_, with lithium layers sandwiched between LiM_2_ layers forming honeycombs (M surrounding Li) [16]. It has also been shown that cations like Li^+^, Mn^2+,^ and Sn^4+^ have a higher mobility in the closely packed oxygen framework, resulting in a substantial amount of intermixing between Li and Sn in the Li_2_SnO_3_ and a highly defected structure [18]. However, a recent work reported the inability of lithium manganate (Li_2_MnO_3_) on direct trapping of CO_2_, although it performs well on CO adsorption [19].

Lithium, sodium, and potassium stannates can be synthesised in different ways resulting in very different sorption performances. Li_2_SnO_3_ can be synthesised by solid-state reactions starting from Li_2_SnOz(OH)_2_, where above 220 °C gradual weight decrease is observed up to 850 °C with sharp endothermic peak around 700 °C linked to the decomposition of Li_2_CO_3_ to Li_2_O and its successive reaction with SnO_2_ to form Li_2_SnO_3_ [20]. Loss of lithium component either by sublimation or reaction with platinum crucible was noticed where heating up to 1000 °C. A temperature of 800 °C and holding time of 4 h at final temperature was found, therefore, to be ideal for Li_2_SnO_3_ synthesis. Despite lithium compounds being easy to sinter, stannates have been known to be hard to sinter. The present Li_2_SnO_3_ was found to have low sinterability even at 1000 °C. The low sintering propension of stannates and the denoted intermixing of Li, Sn ions could represent an advantage in terms of cyclability for CO_2_ adsorption. A study into the function mechanism of CO-CO_2_ atmosphere on the formation of Na_2_SnO_3_ showed the effect of CO_2_ partial pressure on a stannate’s CO_2_ adsorption and desorption capabilities. The adsorption tests show the SnO_2_ surface adsorbed a certain volume of CO_2_ when the SnO_2_ was roasted between 600 °C and 1000 °C [21]. The K-Sn-O system can contain different phases including two Sn^4+^ compounds K_2_SnO_3_ and K_4_SnO_4_, as well as the Sn^2+^ compounds K_2_SnO_2_, K_2_Sn_2_O_3_, and K_4_SnO_3_. Iwasaki et al. (2002) reported the synthesis of new compound Na_4_Sn_3_O_8_ from the reaction of Na_2_CO_3_ and SnO_2_ at 1300 °C [22]. Instead, McAuliffe et al. (2016) synthesised K_2_Sn_3_O_7_ by mixing K_2_CO_3_ and SnO_2_ with molar ratios of 1:1 K:S and calcined the mixture at 900 °C for 9 h in air with heating and cooling rates of 10 °C/min [23]. Nguyen et al. (2016) reported the nucleation and growth of K_2_SnO_3_ nanowires from molten alloy involving KOH and tin oxide in a weight ratio of 1:3 by simple annealing step at 900 °C for 2 h [24].

Due to the requirement to reduce global CO_2_ emissions and to the structural similarities of stannates to zirconates and their lower cost, this work investigates their potential use for CO_2_ capture at high temperatures, since to our knowledge, it has not been explored yet. Therefore, the uptake capacity, adsorption rate, and recyclability of commercial tin (VI) oxide (SnO_2_), zirconium oxide (ZrO_2_), calcium stannate (Ca_2_SnO_3_), sodium stannate (Na_2_SnO_2_), potassium stannate (K_2_SnO_3_), and lithium stannate (Li_2_SnO_2_), were studied by thermogravimetric analysis at different temperatures to evaluate their potential use as high temperature sorbents in industry. Based on its performance, K_2_SnO_3_ was then selected for further study, synthesised in-house using different K:Sn molar ratios, and their CO_2_ sorption capacity and kinetics evaluated and compared to zirconate-based sorbents.

## 2. Results and Discussion

### 2.1. Thermogravimetric Dynamic Analysis

**Figure 1:**
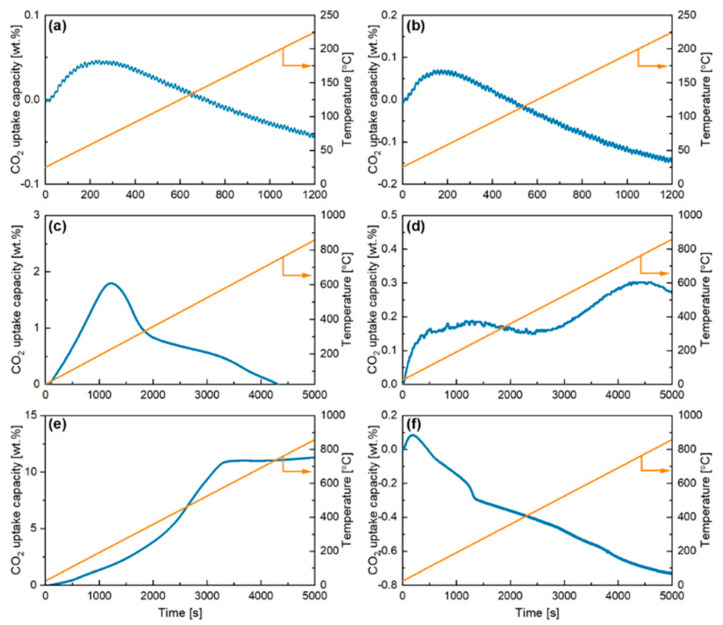
CO_2_ dynamic adsorption for the different commercial sorbents. (**a**) ZrO_2_; (**b**) SnO_2_; (**c**) Na_2_SnO_3_; (**d**) Ca_2_SnO_3_; (**e**) K_2_SnO_3_; (**f**) Li_2_SnO_3_.

The CO_2_ adsorption of tin oxide and zirconium oxide was initially investigated. The TGA analysis results can be seen in Figure 1a,b, while the chemical equations for their reaction with CO_2_ are reported below:

(2) $$ZrO_{2}+2CO_{2}⇌\;Zr(CO_{3)}{}_{2}$$

(3) $$SnO_{2}+2CO_{2}⇌\;Sn(CO_{3)}{}_{2}$$

After an initial desorption of the adsorbed species (not shown in graph) in N_2_, the atmosphere was switched to CO_2_ to begin the sorption process. From the figure, it can be seen that CO_2_ adsorption takes place in the first 200 s for both oxides. Tin and zirconium oxides adsorbed less than 0.1 wt% of CO_2_ before the weight of both samples decreased below their original values. This occurred at low temperature of roughly 40 °C. The derivative graphs for both samples also showed very slow rates for both tin and zirconium oxides (0.0000001 mg/s and 0.00001 mg/s, respectively). As expected, the results indicated that SnO_2_ and ZrO_2_ are not good materials for use in neither high nor low temperature CO_2_ adsorption per se.

After defining a baseline for the support materials, the CO_2_ sorption of commercial Ca, Li, and K stannates was studied as reported in Figure 1c–f. Their respective reaction with CO_2_ is summarised in Equations (4)–(6):

(4) $$CaSnO_{3}+CO_{2}⇌\;SnO_{2}+CaCO_{3}$$

(5) $$Li_{2}SnO_{3}+CO_{2}⇌\;SnO_{2}+Li_{2}CO_{3}$$

(6) $$K_{2}SnO_{3}+CO_{2}⇌\;K_{2}CO_{3}+SnO_{2}$$

Sodium stannate (Figure 1c) resulted in 2 wt% CO_2_ adsorbed in the first 1300 s with peak at around 200 °C and a maximum sorption rate of 0.0011 mg/s. This suggests that Na_2_SnO_3_ is an inefficient sorbent under the studied conditions. For example, a study into the CO-CO_2_ atmosphere on the formation of Na_2_SnO_3_ showed the adsorption of CO_2_ was only favoured at higher partial pressures of CO_2_ above 80 vol% [21]. The analysis of calcium stannate is shown in Figure 1d. This material adsorbed a small amount of CO_2_ (0.3 wt% in total) in two different events taking place at about 30–75 °C due to physisorption and between 500 and 600 °C due to chemisorption with a maximum rate of 0.001 mg/s. The low sorption capacity of the material was unexpected due to previous research into supported calcium sorbents. Typically, CaO is stabilised with the oxides of Al, Mg, and Si whereupon calcination provides excess pore volume allowing CO_2_ particles to enter the particle more easily, thus resulting in higher CO_2_ sorption capacity [25]. There is limited research into the use of stannates for CO_2_ sorption, although a previous report into the formation characteristics of calcium stannate from SnO_2_ and CaCO_3_ found that in a CO-CO_2_ atmosphere the activation energy was 359.97 kJ mol^−1^. Comparing this value to that of sodium zirconate (48.01 kJ mol^−1^) shows how calcium stannate is more dependent on temperature, with the largest reaction found to be at 1100 °C in presence of 85% CO_2_.

Potassium is generally used as a CO_2_ promotor within other ceramics such as Li- or Na-based sorbents or used in low temperature applications. The use of different supports can affect the performance of the potassium sorbent; for example, the use of titanium oxide resulted in the formation of inactive materials thus decreasing the performance [26]. The results from the TGA analysis are shown in Figure 1e, where it can be seen that potassium stannate was found to adsorb the highest amount of CO_2_ out of all the sorbents investigated, with a total of 11.5 wt% adsorbed between ambient temperature and 600 °C and a maximum adsorption rate of 0.0041 mg/s. The weight curve also suggests that the adsorption rate increases at temperatures over 400 °C, which indicates presence of two different sorption mechanisms. The obtained results are summarised in Table 1, where it can be seen that potassium stannate was found to have the best sorption capacities and kinetics.

### 2.2. Performance of Commercial Potassium Stannate under Different Conditions

**Figure 2:**
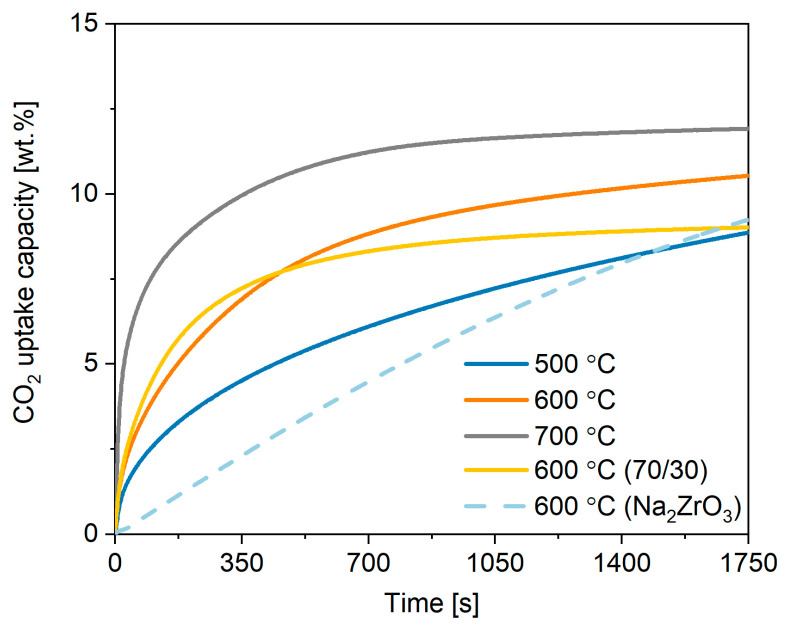
CO_2_ adsorption curves for commercial K_2_SnO_3_ under different temperatures in comparison to Na_2_ZrO_3_ [27], where (70/30) stands for a mixture containing 70 wt.% K_2_SnO_3_ and 30 wt.% Na_2_ZrO_3_.

The commercial potassium stannate was further tested to give an insight into the optimum conditions for its performance. This included investigating the adsorption/desorption temperature, regeneration cycles, and the effect of doping. To investigate the effect of varying the adsorption temperature on the uptake of CO_2_ on the potassium stannate, three different temperatures were analysed: 500, 600, and 700 °C. The results are shown in Figure 2, where it can be seen that carbon dioxide sorption on potassium stannate increased according to the temperature increase with a weight change of 9.3 wt.%, 10.8 wt.%, and 12.2 wt% (2.77 mmol CO_2_/g K_2_SnO_3_) CO_2_ capture respectively at 500, 600, and 700 °C. The CO_2_ sorption rate followed the same trend, with 0.0317, 0.055, and 0.11 (0.18 mg/s) wt.%/min respectively at 500, 600, and 700 ^°^C. If the stannate operates through similar mechanisms to that of sodium zirconate, it can be said that at higher temperatures the primary method of transportation is through the potassium diffusion from the core to the carbonate shell layer after an initial fast surface-driven sorption [27].

Doping is used as a relatively inexpensive method to improve the efficiency of sorbents by combining them with different materials that are known to perform well. The doping effect of combining K_2_SnO_3_ and Na_2_ZrO_3_ at a ratio of 70/30 (wt.%) was investigated and is shown in Figure 2. The doping resulted in the same rate as for the test without Na_2_ZrO_3_ (0.055 wt.%/s) in the first 60 s and a sorption kinetics that levels out after about 500s, resulting in a lower final CO_2_ uptake (9.16 wt.%). Therefore, the addition of sodium zirconate does not seem to be beneficial to the diffusion of the ions through the product layer after the rapid initial CO_2_ sorption has taken place. Sodium zirconate is a well-researched sorbent with a high theoretical sorption capacity and favourable thermodynamics [27]. It has been shown to be effective in high temperature applications with favourable reaction rates. Due to these considerations, it was chosen to be used as a reference material providing a benchmark and a data set against which the stannate-based sorbents could be compared. The results of the TGA analysis can be seen in Figure 2, where the Na_2_ZrO_3_ behaviour at 600 °C under same conditions from previous work was compared [27]. Sodium zirconate has a lamellar structure with the mechanism of adsorption controlled by two processes, the first of which being the alkaline particles reacting with CO_2_ to form the carbonate, and the second being the sodium ions diffusing through the carbonate shell to reach the surface and react with CO_2_ [11]. The uptake and regeneration of Na_2_ZrO_3_ proceeds according to the reversible reaction in Equation (7) with a maximum theoretical increase in weight of 23.75 wt.%.

(7) $$Na_{2}ZrO_{3}+CO_{2}⇌\;Na_{2}CO_{3}+ZrO_{2}$$

The results of the TGA analysis show a total weight increase of 10.3 wt.% CO_2_, at 600 °C with an adsorption rate much slower than that shown by Na_2_SnO_3_. It has to be pointed out that the capacity for sodium zirconate sorbents was found to change with the synthesis, heating rate, and molar ratio of NaCO_3_ and ZrO_2_ reactants having a large effect of the capture performance, so that optimised Na_2_ZrO_3_ can achieve 20–23 wt.% [28]. However, we reported here not-optimised Na_2_ZrO_3_ synthesised under the same conditions for a fair comparison. 

**Figure 3:**
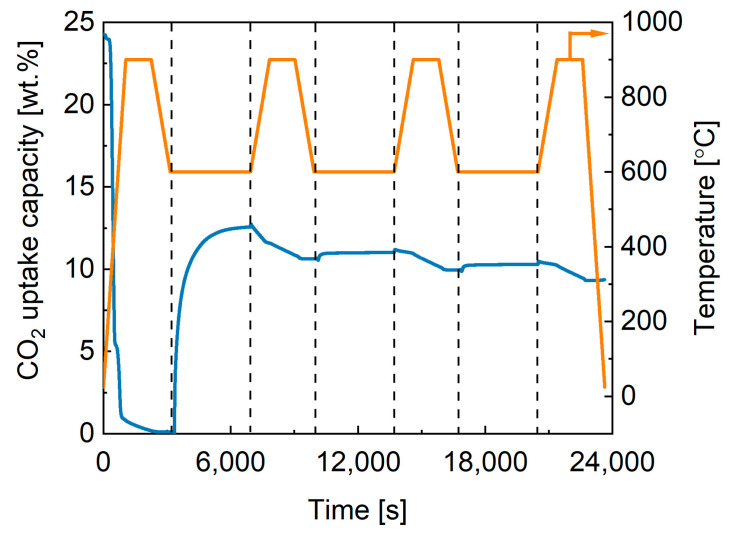
Cyclability test for commercial K_2_SnO_3_ at 600 °C/900 °C.

The regeneration cycle efficiency is an important criterion in sorbent selection as the energy pathway should be low for sorption/desorption to allow the material to be economically useable in industrial settings. A sorbent with a high cyclic stability is preferred where there is a minimal loss of sorption capacity over multiple cycles. Therefore, the commercial potassium stannate was tested over three sorption/desorption cycles with desorption temperature set at 900 °C. As shown in Figure 3, 25 wt.% was released in the initial calcination step, which can be ascribed to the structural water of the trihydrate (18 wt.%) potassium stannate and some additional adsorbed species. Then, in the first sorption step, 12.2 wt% of CO_2_ was adsorbed. However, in the following desorption stage only 2 wt% of CO_2_ was released, suggesting that the commercial material dramatically lost CO_2_ uptake capacity possibly due to sintering or because the temperature used for the synthesis of this material was higher than 900 °C, both ways making the process not viable and energy-intensive. Therefore, a series of K-stannates were synthesised in-house at lower calcination temperature (900 °C) to evaluate its effect on the CO_2_ desorption extent and rate.

### 2.3. Performance of In-House Synthesised Potassium Stannates

**Figure 4:**
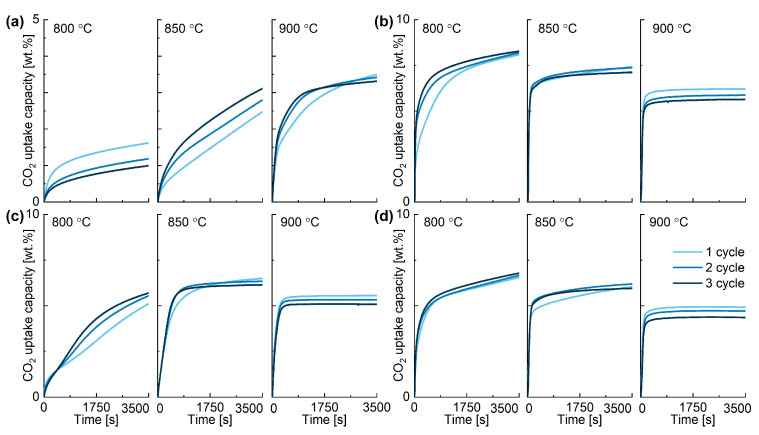
Performance of in-house synthesised potassium stannates (**a**) K-A, (**b**) K-B, (**c**) K-C, (**d**) K-D under different temperatures at 800, 850, 900 °C.

The performance of the in-house synthesised K-stannate sorbents is shown in Figure 4 and summarised in Table 2. The sorbents produced using three different KOH:SnO_2_ weight ratio (K-A 1:2, K-B 3:1, K-C 2:1; K-D 1:1) and a K_2_CO_3_:SnO_2_ wt. ratio of 1:1 (K-D) were evaluated in ten consecutive cycles under three different temperatures (800, 850, and 900 °C), to estimate the CO_2_ uptake capacity, the rates of adsorption and desorption, and the theoretical capacity achieved. The results illustrated in Figure 4 and Table 2 show that K-B resulted in the highest CO_2_ uptake capacity (7.3 wt.% at 800 °C) followed by K-C (6.3 wt.% at 850 °C) and K-D (6.1 wt.% 800 °C) and K-A (2.9 wt.% 900 °C). This reflected the theoretical composition of the three sorbents and their respective theoretical CO_2_ capacities, with the CO_2_ uptake performance following the order K-B > K-D > K-C > K-A at 800 °C and K-B > K-C > K-D> K-A at 850 and 900 °C, respectively. The sorbents perform rather differently changing the temperature from 800 to 900 °C, with K-B and K-D having peak performance at 800 °C, while K-A and K-C showing their best at 900 and 850 °C, respectively. The rate of CO_2_ adsorption-desorption is an important parameter in evaluating CO_2_ sorbents. Among the four synthesised materials, K-B stands out having an adsorption (0.016 mg/s) rate one order of magnitude larger than the other three materials, and desorption (0.016 mg/s) rate comparable with that of K-C at 850 °C. K-B adsorption and desorption rates also are favourable if compared to those obtained under similar conditions by K-orthosilicate (adsorption rate:0.002 mg/s; desorption rate: 0.001 mg/s) [29].

K-B was confirmed to be the most promising high temperature CO_2_ sorbent even in terms of performance based on the maximum theoretical CO_2_ uptake capacity, which was calculated considering the mineral phases quantified from the XRD analysis. K-B high reactivity suggests that the different mineral phase distribution and microstructural differences were behind its better performance. A previous work showed that the use in the solid-state synthesis of lesser amount of KOH than the stoichiometric required resulted in K_2_SnO_3_ nanowires growing on SnO_2_ [24]. However, this was not reflected in the K-A performances, in which initial adsorption rate was not larger than the other sorbents as expected. Another important observation for K-B is that the CO_2_ uptake capacity remained constant in the three cycles under the same temperature (see Figure 4b at 800 °C), suggesting good resistance to sintering at this temperature. Three distinct anhydrous potassium stannates are formed depending on the K and Sn ratio and synthesis conditions used. K_4_SnO_4_ is triclinic, while K_2_SnO_3_ and K_2_Sn_3_O_7_ are orthorhombic with Sn at the centre of a deformed octahedron [30]. It was also found that heating of K_4_SnO_4_ between 650–830 °C converts to K_2_SnO_3_ and then K_2_Sn_3_O_7_ (between 830–900 °C) and into SnO_2_ at higher temperature [31]. The small constant decrease in weight visible in Figure 4 just after the desorption of the CO_2_ in each cycle for K-A and K-B could be attributed to the slow rate transformation of stannate phases, which is also demonstrated by the XRD analysis later discussed.

**Figure 5:**
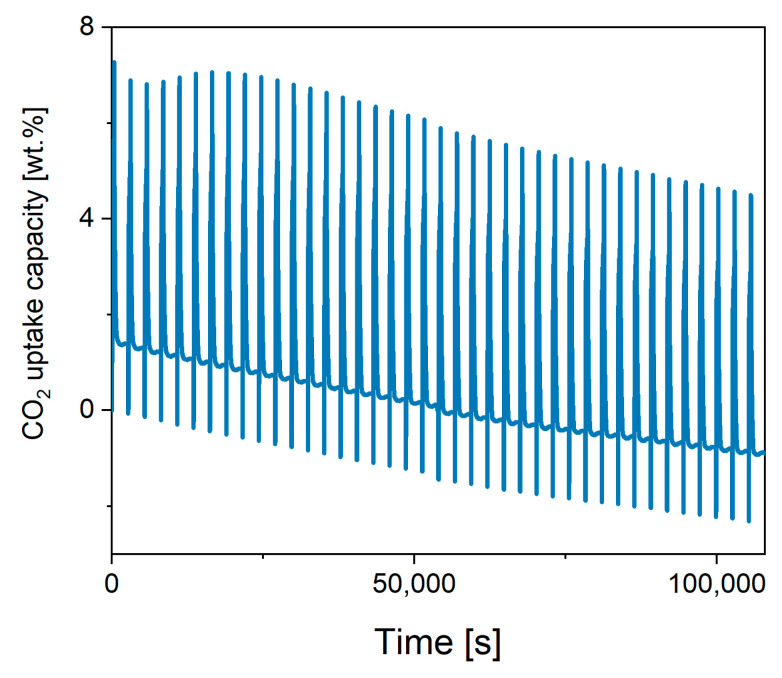
Cyclic stability of K-B after 40 cycles (with both the CO_2_ uptake at 800 °C and regeneration at 900 °C, cycles set at 6 min each, CO_2_ flow 50 mL/min).

**Figure A1:**
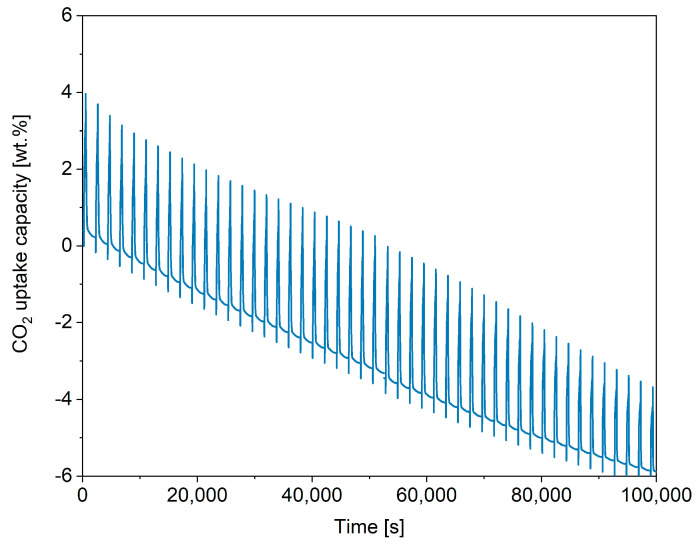
Cyclic stability of K-C after 40 cycles.

**Figure A2:**
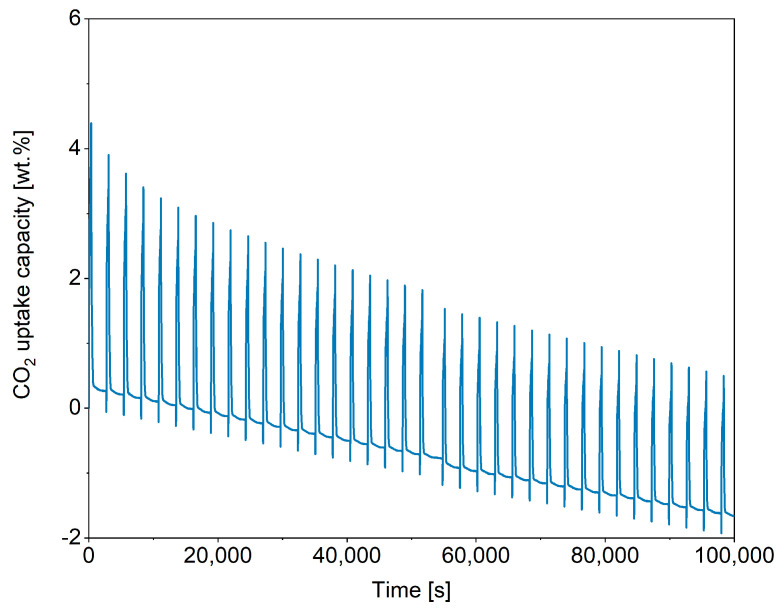
Cyclic stability of K-D after 40 cycles.

The cyclability of high temperature CO_2_ sorbents also represents a paramount aspect for industrial applications, so that a good sorbent should not be prone to deactivation by sintering, or in case of presence of alkali metals, avoid their segregation or sublimation [32]. The TGA results show that both K-B and K-D were stable after three cycles at 800 °C, while all the materials clearly deactivated at 900 °C, suggesting sintering or potassium segregation at that temperature. Interestingly, K-B CO_2_ uptake and cyclability improves cycle after cycle, suggesting that structural/composition changes occur during the process at 800 °C. Therefore, to elucidate this and excluding the low K-A performance, the remnant three stannates’ cyclability was tested as shown in Figure 5, Figure A1 and Figure A2, where the TGA profiles of 40 cycles are reported. The most significant results are also summarised in Table 3. The cyclic stability test for K-B confirmed that an actual CO_2_ uptake capacity gain occurred in the first 10 cycles, which then stabilised with a minor weight capacity loss of 7.4% after 40 cycles. Instead, the CO_2_ uptake capacity of K-C decrease of 17.4% after the same number of cycles and the loss capacity of K-D was even more pronounced, being 52.4%. To put the performance of potassium stannate in context, its capacity uptake was compared to limestone after 50 cycles under similar conditions, where its capacity decreased from more than 45 wt.% to about 7 wt.% after 50 cycles of 30 min at sorption and desorption temperature, respectively, of 650 and 850 °C [33]. Therefore, the main advantage of K_2_SnO_3_ is its stability, while the high desorption temperature represents a drawback shared with CaO, which must be addressed to make the potassium stannate a viable CO_2_ sorbent.

To gain information on the potential reasons behind these differences, XRD, SEM, FTIR, and XPS analyses were carried out.

**Figure 6:**
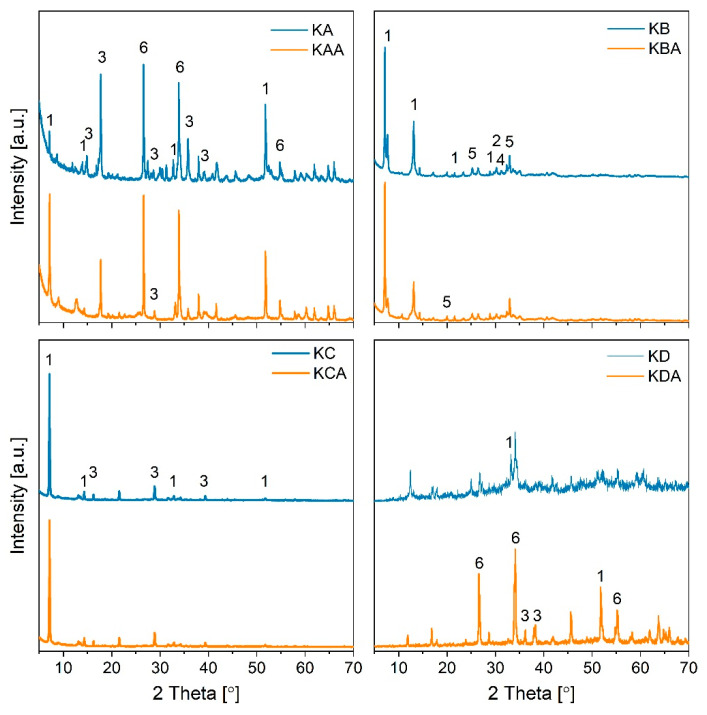
XRD of in-house synthesised sorbents K-A (KA), K-B (KB), K-C (KC), and K-D (KD) and same sorbents after 3 cycles at 850 °C-ads/950 °C-des. 1-K_2_SnO_3_; 2-KOH; 3-K_2_Sn_3_O_7_; 4-K_2_CO_3_; 5-K_4_SnO_4_; 6-SnO_2_.

The samples synthesised using different KOH:SnO_2_ weight ratios present very different XRD profiles (Figure 6). K-A presents about 47% SnO_2_, 34% K_2_Sn_3_O_7,_ and 19% K_2_SnO_3_ phases, where the large abundance of SnO_2_ was expected since KOH was provided in less than the stoichiometric ratio. The K_2_SnO_3_ phase increased to 33% in total peak area after three cycles, while K_2_Sn_3_O_7_ mineral phase decreased to 18% in favour of K_2_SnO_3_, suggesting there is mobility of K ions in the crystalline structure at temperature higher than 800 °C and phase re-arrangement. SnO_2_ diffraction patterns did not suffer any change after 3 cycles. K-C had some similarities with K-B in terms of KOH and K_2_SnO_3_ phase abundance but does not show K_2_Sn_3_O_7_ phase. K-D instead shows an increased crystallinity after the cycles and a composition that somehow resembles that of K-B in terms of K_2_SnO_3_ phase predominance but lacks presence of K_4_SnO_4_ and instead shows K_2_Sn_3_O_7_. These results indicate that the change in rate and uptake capacity visible in Figure 4 between the first and the other two cycles at 850°C can be associated to transformation of K_2_Sn_3_O_7_ in K_2_SnO_3_ with the latter having a faster adsorption rate and capacity (see Equation (8), where theoretical CO_2_ uptake capacity is 8 wt.%).

(8) $$K_{2}Sn_{3}O_{7}+CO_{2}⇌\;K_{2}CO_{3}+SnO_{2}$$

K-B was synthesised with large KOH vs. SnO_2_ weight ratio (4:1) towards the synthesis of K_4_SnO_4_. The XRD patterns do confirm its formation (21%), but large content of K_2_SnO_3_ (76%) was also formed. Unreacted K was also detected together with small amount (3%) of K_2_Sn_3_O_7_. The XRD patterns after three cycles show presence of K_2_CO_3,_ suggesting not complete desorption of CO_2_ during the desorption stages. Also, despite the identified phases not suffering any changes in %, the intensity ratio of the metastannate peaks changed, suggesting a change of its crystals structure. The presence of K_2_SnO_4_ can be linked to the larger CO_2_ uptake capacity of K-B, since one mole of K_4_SnO_4_ can adsorb 2 moles of CO_2_ (see Equation (9)), so that its theoretical CO_2_ uptake capacity (24.8 wt.%) is larger than that of K_2_SnO_3_ (14.7 wt.%).

(9) $$K_{4}SnO_{4}+2CO_{2}⇌\;2K_{2}CO_{3}+SnO_{2}$$

Since the performances of the sorbents could have been affected by other sorbent properties such as surface available, crystal size and crystal type, and basicity, BET, FTIR and XPS analyses were run. The sorbents’ BET surface (m^2^/g) decreased in the order K-D (11.32) > K-B (10.26) > K-A (9.97) > K-C (3.17), indicating that the available surface does not control the CO_2_ uptake capacity and rate, considering that K-B had CO_2_ uptake rate one order of magnitude larger than K-D (See Table 3). Also, the data indicates that the synthesis method can be used to tune the surface available. The crystal size for the different identified phases before and after three cycles at 850 °C was estimated using the Scherrer formula and the results are shown in Table 4.

The first observation is that the crystal size of the different identified mineral phases is consistent among the three adsorbents. Moreover, K_2_SnO_3_ phase crystals in all the three raw samples is around 100–130 nm and the size decreases to about 90 nm after three cycles, suggesting that K_2_SnO_3_ does not suffer sintering. K_2_Sn_3_O_7_ and K_4_SnO_4_ crystals size instead passes from ~60 to ~70/75 nm and 50 to 55 nm, respectively, indicating only a minor increase of the crystal size after 3 cycles. These data confirm the literature on stannates being sintering-resistant [20].

**Figure 7:**
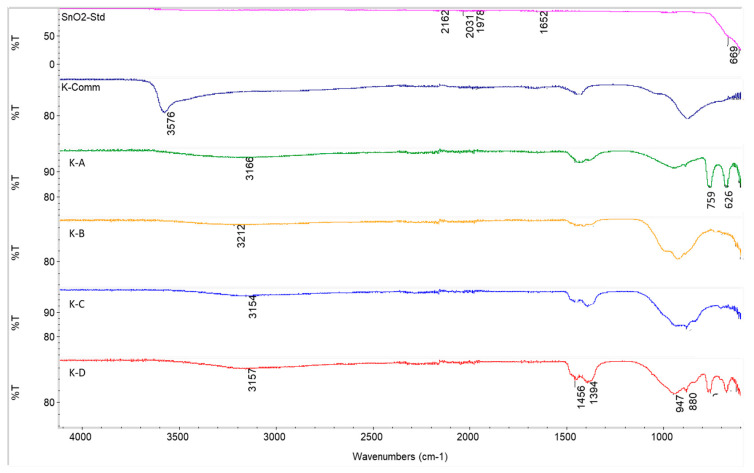
FTIR of tin oxide, commercial and in-house synthesised potassium stannate sorbents.

**Figure 8:**
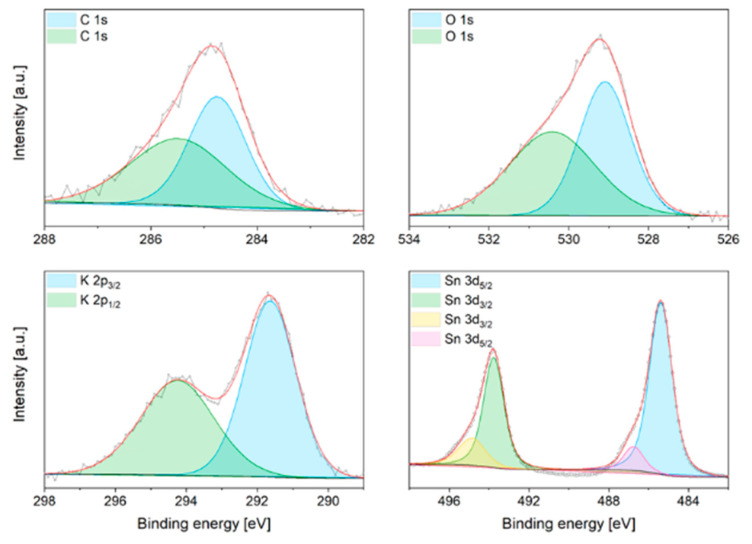
XPS of KB before carbonation.

**Figure A3:**
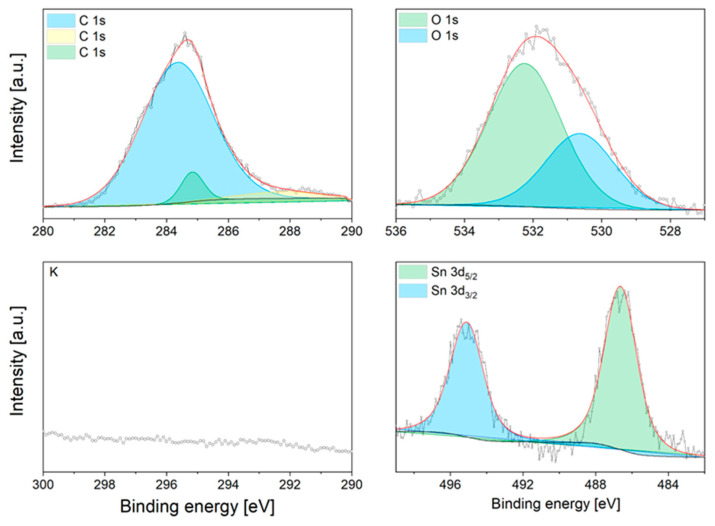
XPS of raw K-A.

Comparing the FTIR profiles of the commercial and in-house synthesised stannate sorbents (Figure 7), it can be clearly seen that while the commercial K-stannate is hydrated, the other ones are not. Another interesting feature is the similarity of K-B and K-C transmittance profiles, which corroborates the similarity (although different distribution) of the mineral phases detected by XRD. The transmittance of the Sn-O bond vibration of SnO_2_ (669 cm^−1^) is well-defined for the K-A sorbent due to the large stoichiometric excess used in the synthesis. The oxidation state of the elements present in the K-stannate sorbents was investigated by XPS. The binding energy (BE) of spin orbital components K2p_3/2_ and K2p_1/2_ was found at 291.8 eV and 294.2 eV and assigned to K ^+1^ in K_2_SnO_3_ and K_4_SnO_4_ as detected by XRD. The 3d spectra of tin (Sn) present in XPS of K-B are shown in Figure 8, where the BE of 3d_5/2_ (485.2 eV) and 3d_3/2_ (493.8 eV) suggests +4 oxidation state. The XPS fitting graph of Sn 3d_5/2_ and Sn 3d_3/2_ showed two best fitted peaks indicating presence of another oxidation state at higher binding energy assigned to Sn^+4^. Similar trend of shift in binding energy was also observed by Ray et al. (2021) [34]. The deconvolution of O1s for K-B revealed two peaks: one at 529 eV was assigned to the O of O-Sn^2+^, while the one at 530.3 eV was assigned to O of O-Sn^4+^. The K-A XPS spectra for Sn3d, O1s, C1s, and K are shown in Figure A3, where it can be distinguished that the Sn3d 5/3 (486.6 eV) and 3/2 of Sn^4+^, the O1s of O-SnO^4+^ (532.2 eV) and O-Sn^2+^ (small peak) at 530.5 eV, confirm large presence of SnO_2_ as expected by the reactant ratio used in the synthesis. K was not detected by the instrument. 

**Figure 9:**
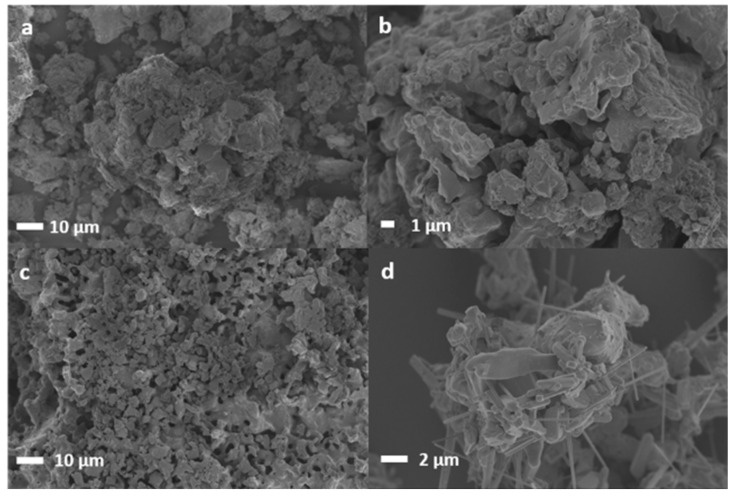
SEM of KB before (**a**,**b**) and after three carbonation/calcination cycles (**c**,**d**).

The surface morphology of K-B before and after carbonation was studied by SEM. Figure 9a K-B showed clear agglomeration of particles, heterogeneity in size distribution, and microporosity. The close-up in Figure 9b shows two types of surfaces: (i) a smooth layer, and (ii) a more irregular surface layer (possibly K-rich) on top of the other layer. Previous work showed similar morphology explaining this superficial layer of K species covering SnO_2_ particles related to the strong philicity of K-O bond of K-species towards the Lewis acidic sites (Sn^4+^-O) present on the surface of SnO_2_ [34]. Figure 9c,d instead shows the morphology of K-B after three carbonation/calcination cycles at different resolutions. The sorbent surface seems made of homogeneous molten phase with presence of macro/mesopores more abundant than the starting one, suggesting some mineral phase rearrangement (as supported by the XRD analysis) by K/Sn/O species migration and temperature-related cracking. Free-moving K^+^ and OH^−^ ions and oxygen radicals produced by high reaction temperature quickly diffuse into tin oxide structure to form molten phase of K_2_SnO_3_/K_4_SnO_4_, which nucleate out in different crystals structures [24]. This is confirmed by the zoomed Figure 9d, also suggesting the presence of different potassium stannate phases, since nanowires, micro sub-spherical, elongated hexagonal prisms, and larger irregular shaped particles are visible. Similar shapes were assigned to K_2_SnO_3_ and K_2_Sn_3_O_7_ by EDX in previous work [34]. K_2_SnO_3_ is a layered structure itself in which the growth predominantly happens at its ends (at edge sites) that can result in nanowires [24]. It can be extrapolated from the SEM pictures that the K-B sorbent was synthesised in the form of fine particles (average size <50 micron). Even though such fine particles can be easily used and tested for characterization in static analysis systems, such as those used in this work, their applicability in actual dynamic systems even at lab-scale, such as fluidized bed reactors, is very challenging. In this context, the use of small sorbent particles can strongly limit sintering and pore-plugging, thus consequently enhancing the multicyclic ability [35]. Moreover, sound-assisted fluidization was proposed as a viable technique to allow the use of such fine particles in fluidized bed reactors, thus overcoming the strict limitation posed by particle size applicable in ordinary fluidized bed reactors [36].

### 2.4. Kinetic Analysis 

**Figure 10:**
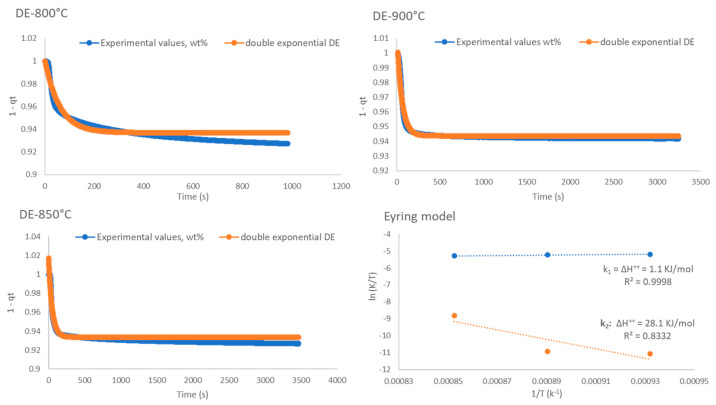
DE and Eyring’s models applied to K-B CO_2_ sorption data.

**Figure A4:**
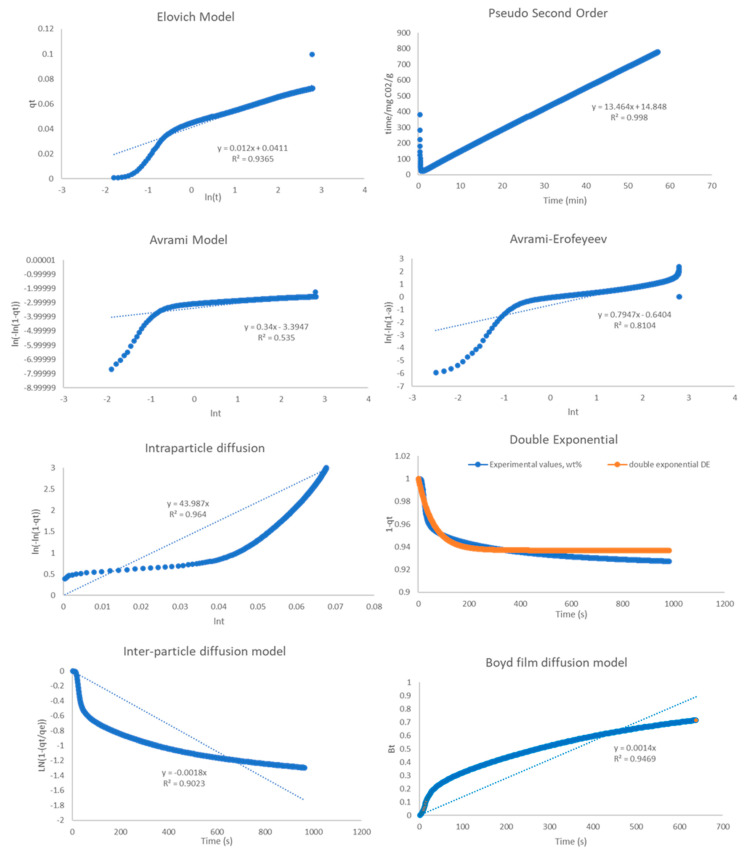
Kinetic modelling at 800 °C.

**Figure A5:**
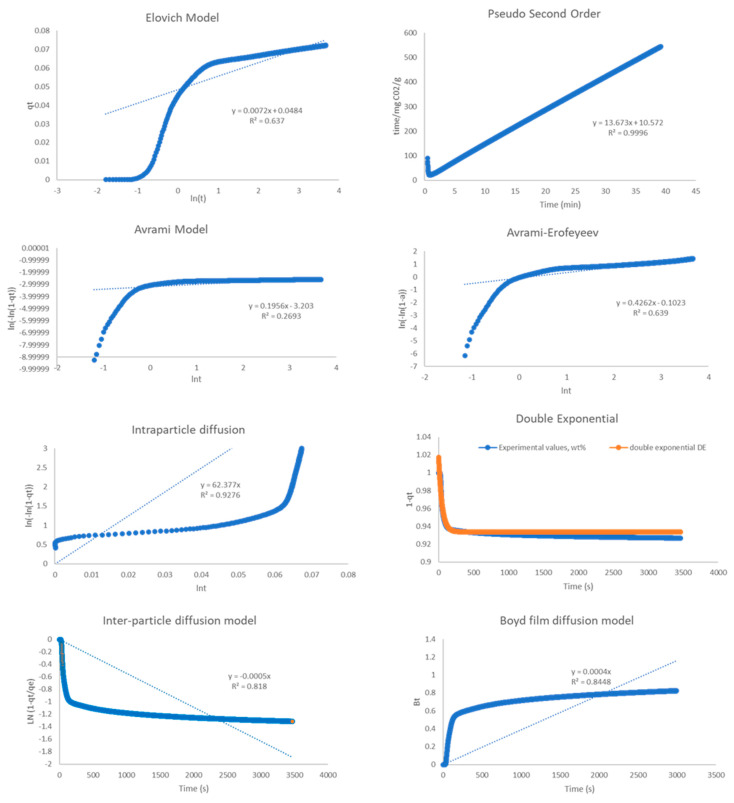
Kinetic modelling at 850 °C.

**Figure A6:**
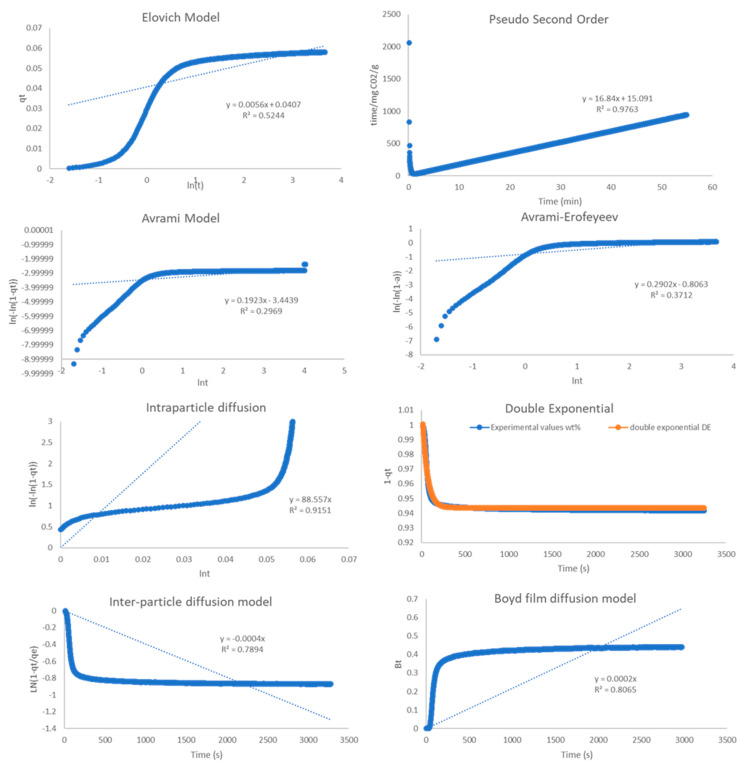
Kinetic modelling at 900 °C.

As visible in Figure 4, the CO_2_ sorption process in presence of K-B can be divided into two steps: (i) a rapid chemical sorption step, and (ii) a slower product layer diffusion. The best model that combines the two phenomena is represented by the double exponential (DE) model, which shows high R^2^ and very good fitting between model and real data in comparison to the other tested models, as shown in Figure 10, Figure A4, Figure A5, Figure A6 and Table 5. Table 6 reports the kinetic parameters calculated with the DE and Eyring’s models. The exponential constant *k*_1_ (chemisorption) was in average 200 times larger than the value of *k*_2_ (bulk diffusion), and the B constants were always larger than A constants and occurred in a large interval of time, indicating that the limiting step of the overall process was potassium diffusion. Furthermore, potassium diffusion increased with the temperature, with *k*_2_ being 10 times larger at 900 °C compared to that at 800 °C. Figure 10 shows the linear plots derived from the Eyring model, where it is possible to see that the superficial and (with less extent) bulk CO_2_ adsorption processes were linear. The calculated activation enthalpies for the surface and bulk reactions were 2.0 and 28.1 kJ/mol. The very low activation enthalpy for the surface chemisorption indicates that this was not dependent on temperature at this range of temperatures. When compared with other sorbents such as Li_4_SiO_4_ [37], the activation enthalpy (∆H ^++^) values of the bulk chemisorption process were greatly reduced, although the temperatures used in that work were lower than in this work. Instead, under similar conditions, the diffusion enthalpy resulted lower in a previous work where Li_4_SiO_4_ was also used, indicating larger diffusion resistance by potassium stannate [38]. On the other hand, the activation entropy (ΔS^++^) was calculated as negative for the surface process (−3.5 kJ/mol) and positive (14 kJ/mol) for the diffusion one, meaning that the entropy for the activated state is lower than the initial state for the surface reaction, which matches with the fact that at the initial state, CO_2_ is in the gas phase.

The apparent DE kinetic models used above indicated the overall adsorption behaviour of K-B and established bulk diffusion of potassium across the carbonate layer formed during the surface chemical reaction as rate-limiting step, but to evaluate the potential presence of other diffusion-limiting steps, the inter-particle, intra-particle diffusion kinetic (IPD) model and the Boyd’s film-diffusion model were used. Applying the inter-particle diffusion model, the plots of ln(1 − qt/qe) versus time exhibited double linearity with poor fitting and the intercept was different from ln6/π^2^ at all temperatures, indicating that inter-particle diffusion was not a rate-limiting step. Using the IPD model and plotting (qt) vs. (t^0.5^) did not result in a good fitting and in a straight line passing through the origin, suggesting that the rate-controlling step was not intra-particle diffusion resistance. Finally, the Boyd’s film-diffusion model also had a poor fitting so that mass transfer controlled by pore-diffusion was excluded.

## 3. Materials and Methods

### 3.1. Materials

Seven different materials were initially selected: Tin (VI) oxide (SnO_2_), zirconium oxide (ZrO_2_), calcium stannate (Ca_2_SnO_3_), potassium stannate trihydrate (K_2_SnO_3_ ·H_2_O), lithium stannate (Li_2_SnO_3_), and sodium stannate trihydrate (Na_2_SnO_3_ ·H_2_O). Tin oxide and zirconium oxide (≥99.99%) were purchased from Sigma-Aldrich (St. Louis, MO, USA) and calcium, potassium, and sodium meta-stannates (≥99%) were acquired from Santa Cruz Biotechnology (Santa Cruz, CA, USA). Then, 4 different K-Sn CO_2_ adsorbents were synthesised in-house and tested. K-A, K-B, and K-C were synthesised by manually mixing (for 5 min in a pestle and mortar) KOH (Sigma-Aldrich) and SnO_2_ (Sigma-Aldrich) using a KOH:SnO_2_ weight ratio of 1:2, 4:1 and 2:1, respectively [24]. The mixture was then calcined in air for 15 hrs at a final temperature set at 900 °C and a heating/cooling rate of 10 °C/min. The reactants’ weight ratios were selected to selectively form: K_2_SnO_3_ nanowires (K-A), K_4_SnO_4_ (K-B), and K_2_SnO_3_ (K-C). The 4^th^ K-Sn material, K-D, was instead synthesised by mixing K_2_CO_3_ and SnO_2_ with molar ratios of 1/1 K/Sn and calcining the mixture at 800 °C for 9 h in air with heating and cooling rates of 10 °C/min towards the production of a K_2_Sn_3_O_7_-rich sample [23].

### 3.2. Experiments

**Figure A7:**
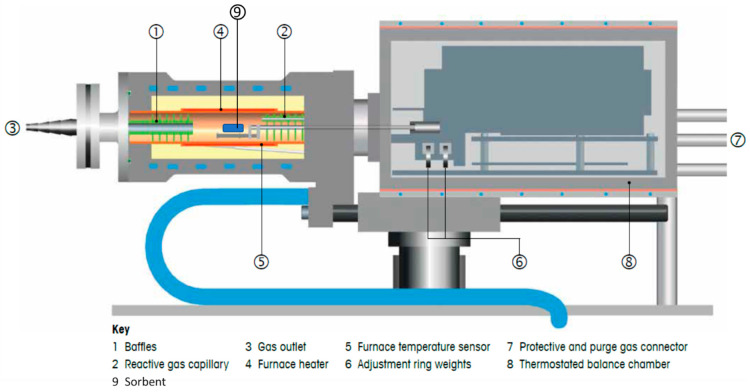
Schematic of TGA2 thermogravimetric balance used for the CO_2_ capture tests. [modified from: https://www.mt.com/dam/Analytical/ThermalAnalysi/TA-PDF/TGA2_Broch_EN_30247078_V04.15_Original_38299.pdf (accessed on 9 November 2022).

CO_2_ uptake capacity and adsorption rates in dynamic environment from 25 to 900 °C under 50 mL/min of N_2_/CO_2_ were evaluated using a Mettler Toledo TGA2 thermogravimetric analyser (Mettler Toledo, Schwerzenbach, Switzerland) depicted in Figure A7. Initially, a small amount of sorbent (~20mg) was placed into the TGA crucible. Pure nitrogen was then passed through the TGA reaction furnace at 100 mL/min until the sample weight became stable. Then, pure CO_2_ was flown in the TG furnace. The sample was heated from 25 to 900 °C at a 10 °C/min. During heating, the sample weight and temperature were continually recorded by computer software and later plotted to analyse the data. Once the maximum temperature was reached, the temperature was kept for 10 min and finally the CO_2_ was desorbed by changing back the atmosphere from CO_2_ to N_2_. The isothermal TGA experiments were run using a method similar to the one described above, with the difference that the samples were first calcined at 900 °C for 15 min in 100% N_2_, then the temperature was decreased to the desired one, equilibrated, and then the N_2_ was switched to CO_2_ for allowing the isothermal CO_2_ adsorption to take place for 5–60 min depending on the experiment. Desorption was then obtained raising the temperature to 900 °C in N_2_. The cyclic performance under three different temperatures (800, 850, and 900 °C) was performed in a sequential way with the same sample. For the cyclic experiments the above steps were repeated 40 times.

### 3.3. Characterisation

X-ray powder diffraction (XRD) was used to inspect the crystalline size, purity, and nature of the sorbents being investigated. The microstructure of the sorbents was analysed before and after the sorption/desorption process, using a Philips PANalytical X’Pert³ XRD, with Kβ filtration by nickel and variable tension and current of 40 kV and 45 mA. The instrument was operated at 25 °C with a Cu anode (K-Alpha1 1.54060 Å with continuous scans between 25 and 100 (°2Th.) with scan step time of 0.5 s. The specimen length was 10 mm and the receiving slit size 0.38 mm. Match! software version 3.15 Build 247 and the COD Inorganics reference pattern database, together with data from literature [31] were used for the identification of the mineral phases. Their relative abundance was estimated using Origin 2019 software by considering the integral relative intensity corresponding to the different identified mineral phases and assuming that this is proportional to the volume fraction in the sample. The analysis of the relative intensity ratio was used for peaks shared by different phases, dividing the most representative peaks by the most intense one. The mean crystallites diameter size (nm) of the Li-FA sorbents was calculated using Scherrer’s formula (D = κλ/βCOSθ), where the Scherrer constant (k) for spherical crystallites with cubic symmetry is equal to 0.9, λ is wavelength (Cu = 1.5406 Å), β is the full width in radian at half maximum (FWHM) of the peaks, and θ is Bragg’s angle of the XRD peak. FTIR analysis was carried out using a PerkinElmer Frontier (PerkinElmer, Waltham, MA, USA) with polarized UATR with incorporated Spectrum 9™ instrument control and data management Spectrum software version 9. X-ray photoelectron spectroscopy (XPS) was used to collect chemical information on the surface of all samples using a PHI Quantera II Scanning XPS Microprobe (Physical Electronics, Chanhassen, MN, USA). Samples were sputtered with argon ions for 30 sec before the measurements. The energy resolved spectra of C, O, Sn, and K were collected and calibrated with C-C peak of C1s. Scanning electron microscopy (SEM) was performed using a Zeiss Merlin Field Emission Scanning Electron Microscope (Zeiss, Oberkochen, Germany) with an acceleration voltage of 2.5 kV. Samples were coated by gold/palladium sputter coater (Polaron SC7640, Thermo VG Scientific, Waltham, MA, USA) for 20 s under 20 mA before performing.

To establish the apparent CO_2_ sorption kinetics of the most performing in-house synthesised sorbent (K-B), the weight change data were fitted to the pseudo-second order, Elovich, Avrami, Avrami-Erofeev, and double exponential kinetic models [20]. In the pseudo-second order model (t/qt) vs. t are plotted as linear function, with slope and intercept representing (1/qe) and (qe^2^/k_2_), respectively. A linear plot of the Elovich model, which links the rate of adsorption to the increase of CO_2_ surface coverage, is obtained plotting (qt) vs. (ln(t)), with slope being (1/β) and intercept (1/β)ln(αβ)). According to the Elovich equation, the activation energy changes linearly with the surface coverage according to E_a_ = E_a_^0^ + ßθ. The Avrami’s fractional order kinetic model of particle nucleation can be applied to CO_2_ chemisorption by plotting −ln(1 − 𝛼)) vs. ln(t), which yields a straight line in the successful fitting of the model. Finally, a linear version of the Avrami-Erofeev kinetic model that predicts gas-solid reactions to follow a growth pattern through nucleation, followed by the subsequent growth of the nuclei that is formed, is obtained plotting ln(−ln(1 − α)) versus ln(t), where if the value of n (curve slope) is higher than 1, the absorption reaction is controlled by the formation and growth of product crystals [27]. Finally, the isotherms were fitted using the Solver function on Excel to a double exponential (DE) model: y =A exp^−k1x^ + B exp^−k2x^ + C where y represents the weight percentage of CO_2_ adsorbed, x is the time, *k*_1_ and *k*_2_ are the exponential factors, which are kinetically controlled by chemisorption and potassium diffusion, and A, B, and C are the pre-exponential factors. The activation enthalpies involved in the above two stages can be calculated using the Eyring absolute rate equation: (ln (k/T) = ln (k_b_/h) + (ΔS^++/^R) + (−ΔH^++/^R) × (1/T), where k_B_ is the Boltzmann’s constant, h is the Planck’s constant, T is the temperature, R is the gas constant, and ΔS^++^ and ΔH^++^ are the activation entropy and enthalpy.

To evaluate then the potential sorption mechanism and rate-controlling steps, the inter-particle, intra-particle, and Boyd’s film-diffusion models were employed. The intra-particle diffusion model, which describes overall adsorption mechanism controlled by film diffusion, external diffusion, surface diffusion, or a combination of these steps, was evaluated by plotting (qt) vs. (t^0.5^), which yields a linear function with (k) and (C) being the slope and intercept, respectively. The Boyd’s film-diffusion model is a single resistance model assuming the gas film surrounding the adsorbent particle is the main resistance. This model can be used to predict the rate-limiting step involved in the adsorption process through the plot of Bt against time t (min). If the plot is straight and passes through the origin, the adsorption rate is controlled by the pore diffusion. However, if the plot is not straight or straight but does not pass through the origin, it can then be deduced that the adsorption process is also controlled by film diffusion [39]. If inter-particle diffusion resistance is the rate-limiting step, a plot of ln(1 − qt/qe) versus t should be linear with the slope of –p(Dc/rp 2) and the intercept should be ln6/p2 [35]. Otherwise, there should be other steps that control the adsorption process.

## 4. Conclusions

The aim of this work was to investigate the use of stannates for carbon capture at high temperatures to help the global effort to reduce CO_2_ emissions. Potassium stannate was found to be a contender for a high temperature CO_2_ sorbent achieving a CO_2_ uptake capacity of 12.2 wt% (2.77 mmol CO_2_/g), which is the highest among K-based sorbents. Also, the CO_2_ uptake rate of the in-house synthesised K-B was one order of magnitude higher than the other sorbents, the uptake was 7.3 wt% after 5 min, and the sorption/desorption cycles stable after 40 cycles. The pseudo-second order model was the most suitable to predict the CO_2_ adsorption process of K-B indicating dominance of chemical adsorption, while film-diffusion resistance and intra-particle diffusion resistance governed the adsorption process in K-B. In summary, this work shows that solid state synthesised potassium stannate could be an effective sorbent for high temperature separation, and further work is required to optimise its synthesis and application, in particular by evaluating its behaviour under different simulated CO_2_ concentrations, presence of moisture, reducing the gap between the current CO_2_ uptake capacity and the theoretical one and decreasing the desorption temperature.

## Figures and Tables

**Table 1:** Summary of stannates’ performance under dynamic CO_2_ adsorption.

		SnO_2_	ZrO_2_	Na_2_SnO_3_	c-K_2_SnO_3_	Ca_2_SnO_3_	Li_2_SnO_3_
Max Mass CO_2_ Adsorbed	(wt%)	0.1	0.1	2.0	11.5	0.3	0.0200
Max Adsorption Rate	(mg/s)	0.0000001	0.00001	0.001	0.0041	0.001	0.0002

**Table 2:** Performance of stannates synthesised in-house.

K-A	Cycle Number (Temperature °C)									
	1 (800)	2 (800)	3 (800)	1 (850)	2 (850)	3 (850)	1 (900)	2 (900)	3 (900)	4 (900)
CO_2_ ads., wt%	1.35	0.78	0.70	2.22	2.63	2.83	3.28	3.17	2.98	2.91
CO_2_ ads, mmol/g	0.31	0.18	0.16	0.51	0.60	0.64	0.75	0.72	0.68	0.66
heor. CO_2_ ads., %	21	12	11	35	41	44	51	50	47	46
CO_2_ des, mmol/g	0.31	0.18	0.16	0.51	0.60	0.64	0.75	0.72	0.68	0.66
CO_2_ ads rate, mg/s	0.0027	0.0019	0.0013	0.0016	0.0022	0.0029	0.0043	0.0053	0.0052	0.0053
CO_2_ des rate, mg/s	0.0021	0.0018	0.0016	0.0044	0.0084	0.01	0.0094	0.0086	0.0081	0.0074
	Cycle number (Temperature °C)									
K-B	1 (800)	2 (800)	3 (800)	1 (850)	2 (850)	3 (850)	1 (900)	2 (900)	3 (900)	4 (900)
CO_2_ ads., wt%	7.02	7.29	7.32	6.52	6.69	6.48	5.29	4.95	4.74	4.27
CO_2_ ads, mmol/g	1.59	1.66	1.66	1.48	1.52	1.47	1.20	1.13	1.08	0.97
Theor. CO_2_ ads., %	46	48	48	43	44	43	35	33	31	28
CO_2_ des, mmol/g	1.59	1.64	1.63	1.52	1.50	1.46	1.35	1.20	1.11	1.04
CO_2_ ads rate, mg/s	0.0062	0.016	0.018	0.012	0.016	0.016	0.009	0.008	0.008	0.008
CO_2_ des rate, mg/s	0.017	0.012	0.011	0.015	0.016	0.015	0.019	0.018	0.017	0.017
K-C	Cycle number (Temperature °C)									
	1 (800)	2 (800)	3 (800)	1 (850)	2 (850)	3 (850)	1 (900)	2 (900)	3 (900)	4 (900)
CO_2_ ads., wt%	4.88	5.21	5.25	6.14	5.98	6.29	5.16	4.73	4.64	4.39
CO_2_ ads, mmol/g	1.11	1.19	1.19	1.40	1.36	1.43	1.17	1.08	1.06	1.00
Theor. CO_2_ ads., %	37	40	40	47	45	48	39	36	35	33
CO_2_ des, mmol/g	1.11	1.19	1.19	1.40	1.36	1.43	1.17	1.08	1.06	1.00
CO_2_ ads rate, mg/s	0.0051	0.0043	0.0026	0.0031	0.0037	0.0031	0.0092	0.007	0.0063	0.0061
CO_2_ des rate, mg/s	0.0091	0.0098	0.01	0.022	0.022	0.022	0.026	0.025	0.024	0.024
K-D	Cycle number (Temperature °C)									
	1 (800)	2 (800)	3 (800)	1 (850)	2 (850)	3 (850)	1 (900)	2 (900)	3 (900)	4 (900)
CO_2_ ads., wt%	5.91	5.99	6.14	5.64	5.73	5.38	4.51	4.08	3.88	3.60
CO_2_ ads, mmol/g	1.34	1.36	1.40	1.28	1.30	1.22	1.03	0.93	0.88	0.82
Theor. CO_2_ ads., %	45	45	47	43	43	41	34	31	29	27
CO_2_ des, mmol/g	1.34	1.36	1.39	1.28	1.30	1.22	0.98	0.90	0.84	0.77
CO_2_ ads rate, mg/s	0.0051	0.0043	0.0026	0.0031	0.0037	0.0031	0.0092	0.007	0.0063	0.0061
CO_2_ des rate, mg/s	0.0091	0.0098	0.01	0.022	0.022	0.022	0.026	0.025	0.024	0.024

**Table 3:** Performance of K-B, K-C, and K-D under long exposition (with both the CO_2_ uptake at 800 °C and regeneration at 900 °C, cycles set at 360 s each, CO_2_ flow 50 mL/min).

	K-B				
Cycle	1	10	20	30	40
CO_2_ ads., wt.%	5.78	6.28	5.70	5.65	5.35
% lost	0	−8.7	1.4	2.2	7.4
	K-C				
Cycle	1	10	20	30	40
CO_2_ ads., wt.%	3.44	3.30	3.32	3.31	2.84
% lost	0	4.1	3.5	3.8	17.4
	K-D				
Cycle	1	10	20	30	40
CO_2_ ads., wt.%	3.55	2.29	2.16	1.88	1.69
% lost	0	35.5	39.2	47.0	52.4

**Table 4:** Crystal size estimation from Scherrer formula.

Sample	Peak Position 2θ (°)	FWHM B_size_ (°)	Dp (nm)
KA-K_2_SnO_3_	51.77	0.15	102.85
KA-K_2_Sn_3_O_7_	17.72	0.2	59.60
KA-SnO_2_	26.6	0.16	84.48
KAA-K_2_SnO_3_	51.77	0.16	91.38
KAA-K_2_Sn_3_O_7_	17.72	0.17	75.71
KAA-SnO_2_	26.6	0.15	93.76
KB-K_2_SnO_3_	7.15	0.13	117.18
KB-K_4_SnO_4_	32.94	0.23	50.64
KBA-K_2_SnO_3_	7.15	0.15	91.43
KBA-K_4_SnO_4_	32.94	0.216	55.15
KC-K_2_SnO_3_	7.15	0.11	126.04
KC-K_2_Sn_3_O_7_	29.46	0.18	70.96

**Table 5:** R^2^ for the various kinetic models evaluated for K-B.

Model	R^2^		
	800 °C	850 °C	900 °C
Pseudo-2nd order	0.999	0.999	0.976
Elovich	0.941	0.637	0.524
Avrami	0.534	0.269	0.297
A-E	0.81	0.639	0.371
Double Exp.	0.975	0.996	0.944
Intra-P Diffusion	0.964	0.928	0.915
Inter-P Diffusion	0.902	0.818	0.789
Boyd Film Diff.	0.947	0.845	0.806

**Table 6:** Kinetic parameters obtained applying the DE model.

Double Exponential Model						Eyring’s Model		ln(k_1_/T)	ln(k_2_/T)
T, K	k_1_, s^−1^	k_2_, s^−1^	A	B	C	T, K	1/T	k_1_	k_2_
1073	4.8870	0.0168	0.01	0.06	0.94	1073	0.0009	−5.392	−11.065
1123	5.9870	0.0203	0.01	0.09	0.93	1123	0.0009	−5.234	−10.923
1173	6.2270	0.1762	0.00	0.07	0.97	1173	0.0009	−5.238	−8.8035

## Data Availability

Data will be made available on request.
